# Daily Ischemic Preconditioning Provides Sustained Protection From Ischemia–Reperfusion Induced Endothelial Dysfunction: A Human Study

**DOI:** 10.1161/JAHA.112.000075

**Published:** 2013-02-22

**Authors:** Mary Clare Luca, Andrew Liuni, Kelsey McLaughlin, Tommaso Gori, John D. Parker

**Affiliations:** 1 Division of Cardiology, Mount Sinai and University Health Network Hospitals, Toronto, Canada (M.C.L., A.L., K.M.L., J.D.P.); 2 Department of Pharmacology and Toxicology, University of Toronto, Canada (M.C.L., A.L., K.M.L., J.D.P.); 3 Medizinische Klinik, University Medical Center Mainz, Germany (T.G.)

**Keywords:** cyclooxygenase‐2, endothelium, ischemia, ischemic preconditioning, reperfusion

## Abstract

**Background:**

It is well established that acute ischemic preconditioning (IPC) protects against ischemia–reperfusion (IR) injury; however, the effectiveness of repeated IPC exposure has not been extensively investigated. We aimed to determine whether daily IPC episodes provide continued protection from IR injury in a human forearm model, and the role of cyclooxygenase‐2 in these responses.

**Methods and Results:**

Thirty healthy volunteers were randomized to participate in 2 of 3 protocols (IR alone, 1‐day IPC, 7‐day IPC) in an operator‐blinded, crossover design. Subjects in the IR alone protocol underwent flow‐mediated dilation (FMD) measurements pre‐ and post‐IR (15′ upper‐arm ischemia and 15′ reperfusion). The 1‐day IPC protocol involved FMD measurements before and after 1 episode of IPC (3 cycles of 5′ upper‐arm ischemia and 5′ reperfusion) and IR. Day 7 of the 7‐day IPC protocol was identical to the 1‐day IPC protocol but was preceded by single daily episodes of IPC for 6 days prior. During each protocol, subjects received a 7‐day treatment of either the cyclooxygenase‐2 inhibitor celecoxib or placebo. Pre‐IR FMD was similar between groups. IR alone reduced FMD post‐IR (placebo, ΔFMD: −4.4±0.7%; celecoxib, ΔFMD: −5.0±0.5%). One‐day IPC completely prevented this effect (placebo, ΔFMD: −1.1±0.6%; celecoxib, ΔFMD: 0.0±0.7%; *P*<0.0001). Similarly, 7‐day IPC demonstrated persistent endothelial protection post‐IR (placebo, ΔFMD: −0.9±0.9%; celecoxib, ΔFMD: 0.0±0.8%; *P*<0.0001, *P*<0.0001 for ANOVA effect of IPC protocol). Celecoxib did not alter responses to IR in any protocol.

**Conclusions:**

Daily episodes of IPC provide sustained protection from IR‐induced endothelial dysfunction in humans through a mechanism that appears cyclooxygenase‐2‐independent.

## Introduction

Restoration of blood flow after severe ischemia or ongoing infarction results in further tissue damage, a phenomenon known as ischemia–reperfusion (IR) injury. IR injury has been shown to cause arrhythmias, vascular endothelial dysfunction, myocardial stunning, and cell death.^1^ Endothelial cells are particularly sensitive to, and actively participate in the progression of IR injury. They are the first cellular population to experience reperfusion and have a major impact on subsequent blood flow responses to areas of prior ischemia.^2–3^ A state of endothelial dysfunction commonly occurs following IR injury, which can impair tissue perfusion through the activation of neutrophils and platelets and exacerbate tissue damage.^4^ The endothelium's sensitivity to IR injury has been demonstrated in several experiments where exposure to short periods of IR caused selective endothelial dysfunction while leaving vascular smooth muscle function unimpaired,^2,4^ although this has not been consistently demonstrated.^5^ Furthermore, it has been shown that the kinetics of cell death during IR are cell‐specific with endothelial cells succumbing prior to cardiomyocytes.^6^ Thus, the endothelium is extremely sensitive to IR injury, but at the same time is a major determinant of the tissue's ability to recover from IR. Therefore, studies aimed at protecting the endothelium from IR, particularly when conducted in humans, should be considered of direct clinical interest.

It has been demonstrated that exposure to brief periods of ischemia (termed ischemic preconditioning [IPC]) before a prolonged ischemic insult can reduce myocardial and endothelial sensitivity to IR‐induced injury,^4,7^ a phenomenon that has been observed in every species tested, including humans.^8^ The potent protective effects of preconditioning provide attractive opportunities for therapeutic intervention. For example, preconditioning may have clinical applications in the setting of programmed ischemia (eg, during percutaneous coronary angioplasty^9^ or coronary artery bypass surgery^10^). Although preconditioning interventions have been used to improve mortality and morbidity in patients experiencing spontaneous myocardial infarction,^11^ this therapeutic approach remains largely unexploited in clinical practice. An important, intrinsic limitation is that spontaneous, acute myocardial infarction therapeutic preconditioning can only be applied after the onset of infarction, an approach that limits their effectiveness as compared to animal models of “planned” myocardial infarction. One approach to this problem, which might find application in patients at high risk of myocardial infarction, would be the development of repeated preconditioning treatment algorithms that would provide ongoing protection from the effects of IR. To date, this approach has not been explored in humans. Therefore, the purpose of the current investigation was to determine whether the protective effects of IPC are maintained when administered repeatedly over time.

## Methods

### Population

Thirty young, healthy, male volunteers (20 to 31 years old) were enrolled to participate in this operator‐blinded, placebo‐controlled, crossover design study. The Mount Sinai Research Ethics Board approved the protocols and all subjects gave informed consent prior to beginning the study. Exclusion criteria included any active disease, the use of medications (including supplemental vitamins), as well as risk factors for cardiovascular disease such as hypertension, smoking, or hypercholesterolemia.

### Study Protocols

**Figure 1.**
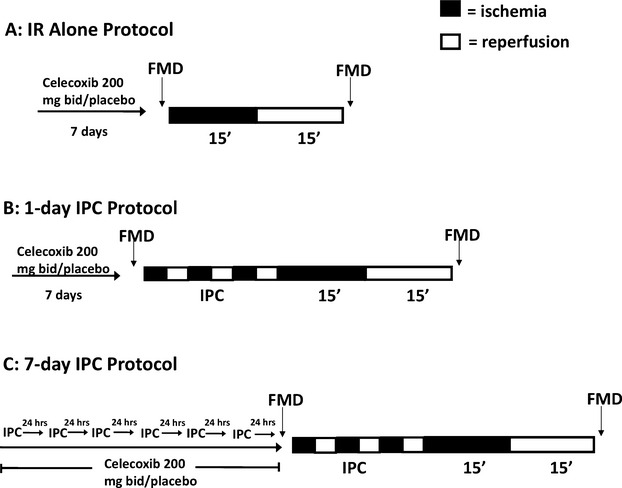
A, IR alone protocol. After a 7‐day treatment of celecoxib 200 mg bid or matching placebo, subjects underwent assessments of FMD before and after IR. n=10 per treatment. B, One‐day IPC protocol. After a 7‐day treatment of celecoxib 200 mg bid or matching placebo, subjects underwent assessments of FMD before and after IPC‐IR. n=10 per treatment. C, Seven‐day IPC protocol. Subjects visited the laboratory on 6 consecutive days to undergo a single episode of IPC, simultaneous with celecoxib 200 mg bid or matching placebo treatment. On the seventh day, subjects underwent assessments of FMD before and after IPC‐IR. n=10 per treatment. IPC indicates ischemic preconditioning; FMD, flow‐mediated dilation; IR, ischemia–reperfusion.

This study involved 3 separate protocols, which were designed to study the effect of (1) IR alone on flow‐mediated dilation (FMD) (with no IPC); (2) the effect of IR on FMD after a single episode of IPC and, finally (3) the effect of IR on FMD after 7 days of repeated episodes of IPC. Each protocol included 20 volunteers randomized (in a double‐blind fashion) to either celecoxib 200 mg bid or matching placebo bid. Each participant participated in 2 of the 3 protocols (Figure 1).

### Study Methodology

Studies were conducted in a quiet, temperature‐ and humidity‐controlled environment. All subjects were required to fast and abstain from caffeine for 24 hours prior to the study. After admission to the study, subjects were randomized in a double‐blind fashion to celecoxib 200 mg bid or placebo. They were subsequently randomized to participate in 2 of 3 protocols (IR alone, 1‐day IPC, 7‐day IPC; described below) in a crossover fashion (with a minimum 7 days of washout between each protocol). A research nurse not involved in acquisition or analysis of study data performed all randomization and maintained appropriate logs. The first author performed the preconditioning protocols including data acquisition and measurement. Upon completion of the study, data files were coded and all FMD analyses were performed in a blinded fashion.

### IR Alone Protocol

Initially, subjects were randomized to 200 mg bid of celecoxib or matching placebo for the 7 days prior to, and including the day of the study visit. On the day of the study visit, standing blood pressure measurements were obtained after 10 minutes of rest, using an automatic, calibrated sphygmomanometer (GE Healthcare). Radial artery FMD was then measured as previously described.^12–14^ Subsequently, subjects underwent an episode of forearm IR by placing a pneumatic cuff at the level of the brachial artery and inflating it to 250 mm Hg for 15 minutes, followed by 15 minutes of reperfusion. FMD measurements were repeated immediately following the period of IR (Figure 1A). This model, which causes a marked impairment in endothelium‐dependent vasodilation while leaving endothelium‐independent vasodilation intact,^4^ has been recently employed in multiple studies of IR injury and preconditioning (including a number from our group).^12–14^ The repeatability and reproducibility of FMD as assessed in our laboratory have been described previously.^15^

### 1‐Day Protocol

The 1‐day protocol consisted of subjects being administered 200 mg bid of celecoxib or matching placebo for the 7 days prior to and including the day of the study visit. On the day of the study visit, subjects underwent a baseline assessment of FMD followed by a single episode of IPC (3 cycles of 5′ upper arm ischemia followed by 5′ reperfusion). Subsequently, subjects underwent the 15′ ischemia and, after 15′ of reperfusion, a second assessment of FMD (Figure 1B).

### 7‐Day Protocol

The 7‐day protocol consisted of subjects visiting the laboratory 6 consecutive times, each 24 hours apart, during which they underwent a single episode of IPC as described above. On day 1, subjects began a 7‐day regimen of 200 mg bid of celecoxib or matching placebo. Twenty‐four hours after the end of the sixth IPC visit, the seventh visit consisted of measuring radial artery FMD before and after episodes of IPC and IR as in the 1‐day protocol (Figure 1C).

### Statistical Analysis

Sample size estimates for this study assume 1−β=0.8 and a 2‐sided α of 0.05. For this study, sample size estimates were made based on previous data from our laboratory.^12^ IR decreased FMD responses from 7.9±3.3% to 1.2±2.3%. Prevention of 50% of this impairment via IPC‐induced preconditioning requires a sample size of 10 subjects in each crossover group. A 2‐way analysis of variance was carried with the different IPC protocols (ie, IR alone, 1‐day IPC, or 7‐day IPC) and drug (placebo or celecoxib) as between‐subject factors (IPC protocol and drug, respectively). This analysis was used to assess the effects of both IPC and drug allocation on the change in FMD after IR (ΔFMD) in the 3 protocols. Post hoc comparisons were performed using the Bonferroni correction. A value of *P*<0.05 was set as the threshold for significance. All results are presented as mean±SE. SAS 9.1.3 (SAS Institute, Inc.) was used for all statistical analyses.

## Results

**Figure 2.**
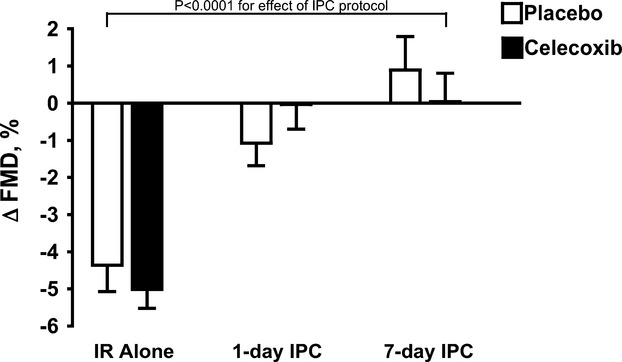
Change in the FMD response with IR (ΔFMD) in IR alone, 1‐day IPC and 7‐day IPC groups treated with placebo and celecoxib. n=10 per group. IR indicates ischemia–reperfusion; FMD, flow‐mediated dilation; IPC, ischemic preconditioning.

Radial artery diameter, the change in radial artery diameter in response to reactive hyperemia (ie, during FMD), and blood flow data are presented in the Table. ΔFMD data are presented in Figure 2.

### Baseline Parameters

There were no significant differences in radial artery diameter, baseline blood flow, reactive hyperemia or change in arterial diameter following wrist cuff deflation between protocols in those were treated with placebo or celecoxib (Table 1). Hemodynamic parameters (systolic blood pressure, diastolic blood pressure, and mean arterial pressure) were not significantly different between any of the groups.

**Table 1.** Arterial Diameter and Blood Flow Data

Radial Artery Diameter, mm	Before IR		After IR	
	Baseline Diameter	Absolute Change in Diameter After Wrist Cuff Deflation	Baseline Diameter	Absolute Change in Diameter After Wrist Cuff Deflation
Placebo				
IR alone	2.23±0.13	0.17±0.01	2.21±0.13	0.07±0.01*
1‐day IPC	2.42±0.09	0.16±0.01	2.40±0.10	0.13±0.02
7‐day IPC	2.41±0.12	0.19±0.02	2.42±0.13	0.20±0.02
Celecoxib				
IR alone	2.26±0.12	0.17±0.02	2.24±0.12	0.06±0.01*
1‐day IPC	2.34±0.09	0.16±0.02	2.26±0.08	0.15±0.02
7‐day IPC	2.27±0.10	0.16±0.02	2.25±0.10	0.16±0.02

Blood Flow, mL/min	Before IR			After IR		
	Baseline	After Wrist Cuff Deflation	Reactive Hyperemia, %	Baseline	After Wrist Cuff Deflation	Reactive Hyperemia, %
Placebo						
IR alone	12.4±4.3	87.3±15.0	1080±203	8.7±1.9	80.7±12.6	929±111
1‐day IPC	15.3±3.6	119.5±12.9	1003±228	12.3±2.0	109.4±10.3	937±146
7‐day IPC	13.4±3.4	103.7±14.3	941±158	11.0±3.8	101.6±15.4	1094±224
Celecoxib						
IR alone	11.0±2.2	89.3±10.8	879±152	11.4±3.1	89.4±13.5	1060±218
1‐day IPC	13.4±1.7	110.7±13.1	793±127	10.7±2.8	102.8±11.9	1340±432
7‐day IPC	13.0±4.1	110.0±19.5	912±203	11.5±4.7	101.0±17.3	1064±230

Radial artery diameter and blood flow at baseline and during reactive hyperemia are presented. n=10 per group. IR indicates ischemia–reperfusion; IPC, ischemic preconditioning.

* *P<*0.001; **P<0.0001: both for absolute change from baseline compared to before IR.

### Effect of IR

There were no significant differences in either baseline diameter or baseline blood flow before versus after IR in any protocol (*P*=NS for all; Table), nor were there any differences in peak reactive hyperemia before versus after IR in any of the protocols (Table). In both the placebo and celecoxib groups, IR alone reduced FMD (Figure 2; placebo, ΔFMD: −4.4±0.7%; celecoxib, ΔFMD: −5.0±0.5%), whereas the adverse effect of IR on FMD with IR was significantly reduced in both the 1‐day IPC (Figure 2; placebo, ΔFMD: −1.1±0.6%; celecoxib, ΔFMD: 0.0±0.7%; *P*<0.0001) and 7‐day IPC protocols (Figure 2; placebo, ΔFMD: −0.9±0.9%; celecoxib, ΔFMD: 0.0±0.8%; *P*<0.0001). The ANOVA revealed a significant effect of IPC protocol on ΔFMD (*P*<0.0001). There was no difference in the protective effect of IPC between the 1‐day and 7‐day IPC protocols.

### Effect of Celecoxib

In each of the protocols (IR alone, 1‐day IPC, 7‐day IPC), there were no significant differences between the placebo and celecoxib cohorts with regards to baseline parameters (ie, hemodynamic parameters, or baseline diameter, baseline blood flow, reactive hyperemia, and FMD before IR), nor were there any differences in the FMD responses with IR (*P*=NS for all; Table and Figure 2).

## Discussion

Since the initial observation that brief, sublethal episodes of myocardial ischemia provide remarkable protection in the setting of myocardial function,^7^ the phenomenon of IPC has been shown to be reproducible and effective in multiple organs and species. IPC has generated tremendous scientific interest being described as the most powerful available form of in vivo protection against myocardial ischemic injury.^16^ However, despite over 20 years of research, preconditioning strategies have not yet been widely implemented in clinical practice. Most efforts in the development of therapeutic cardiovascular preconditioning have been designed to limit damage in the setting of acute myocardial infarction. In this case, preconditioning (or “perconditioning”) stimuli are used in an effort to limit damage after the onset of the infarction process. Preconditioning interventions have also been used in an effort to limit damage during procedures where IR injury predictably occurs (such as during percutaneous interventions and cardiopulmonary bypass).^17^ Theoretically, the phenomena of preconditioning could also be used in patients to limit symptoms (eg, exertional angina)^18^ or to provide ongoing protection from the consequences of acute ischemia or infarction. The ability to exploit this potential clinical application of preconditioning (either ischemic or pharmacologic) is dependent on whether a “preconditioned state” can be maintained over time. With these considerations in mind, the development of preconditioning techniques that do not result in tachyphylaxis or tolerance when administered in a repetitive fashion is an important therapeutic goal. A number of studies have examined the ability of repeated pharmacologic stimuli to induce a sustained preconditioning phenotype. Exposure to HMG‐CoA reductase inhibitors, opioid agonists, chronic adenosine receptor activation, and nitroglycerin have been mixed, with some experiments demonstrating sustained protection with others showing loss of protection over time.^19–22^ Although a single exposure to nitroglycerin has repeatedly been shown to have protective cardiovascular effects in humans,^9,13^ we recently reported that repeated, daily 2‐hour exposure to nitroglycerin was associated with loss of its preconditioning effects.^23^ HMG‐CoA reductase inhibitors have also been explored as preconditioning agents in models of human disease. A number of studies have documented that HMG‐CoA reductase inhibitors are protective in the setting of angioplasty, myocardial infarction, and cardiac surgery.^24–27^ Although these studies made use of sustained dosing regimens, the positive treatment effect was driven mostly by an impact on periprocedural events and the independent impact of “chronic dosing” was not clearly defined. Our group has recently completed a study in which the effects of 3 weeks of daily therapy with rosuvastatin on the response to IR were compared to those observed in a placebo group. In contrast to our previous report in which a single exposure to rosuvastatin had pronounced preconditioning effects, sustained exposure to rosuvastatin provided no protection from IR injury.^28^

Remarkably, the concept of inducing sustained preconditioning with repeated ischemic stimuli over time has received less attention. Recent studies employing a swine model of myocardial infarction compared a classic short‐term IPC stimulus to both repeated episodes of transient low flow ischemia and repeated transient coronary occlusions, both administered every 12 hours over a 72‐hour period.^29–30^ These investigations found that repeated episodes of IPC over this 3‐day period were associated with sustained protection from the effects of IR. These authors also reported that the mechanism(s) of preconditioning protection from single versus repeated episodes of IPC were different. The effect of single episodes of IPC was dependent upon an upregulation in NO synthesis and could be blocked by a NO synthase inhibitor, however, repeated episodes of IPC were not.^30^ These findings are in contrast with an earlier report of a rabbit model of IR injury, where very frequent IPC stimuli (40 to 65 periods of IPC over a period of 3 to 4 days), resulted in a complete loss of cardioprotection as compared to a single IPC episode.^31^

In humans, only limited information is available concerning the effects of repeated preconditioning. To date, no human study has reported the impact of repeated, controlled IPC stimuli on the effect of IR. One prospective study, using self‐reported episodes of angina as a surrogate for IPC, showed that multiple episodes (ie, >4) of angina in the 48 hours prior to the incidence of myocardial infarction resulted in a similar level of cardioprotection as that observed in patients who had experienced a single episode during that time period.^32^ This observation suggests that the myocardium does not become tolerant or insensitive to the effects of IPC when induced endogenously through multiple episodes of angina prior to prolonged coronary ischemia.

The present investigation demonstrates that 7 episodes of IPC, repeated daily, confer protection against IR‐induced endothelial damage in the radial artery that is equal to that conferred by a single episode of IPC. Further, our results suggest that the protection provided by both single and repeated episodes of IPC are not mediated by a mechanism that involves COX‐2, as the preadministration of the selective COX‐2 inhibitor celecoxib did not alter the effect of IPC. However, as mentioned above, COX‐2 has been shown to be an important mediator in the delayed IPC response. Several animal studies have demonstrated that cardioprotection afforded by delayed IPC is associated with an upregulation of the COX‐2 protein 24 hours after IPC, along with an increased content of the by‐products of COX‐2 activity: prostaglandin E_2_, 6‐keto‐prostaglandin F_1α_.^33^ These prostaglandins are known to have independent protective effects in the setting of IR.^34^ This activity of COX‐2 has been shown to be regulated by the inducible isoform of nitric oxide synthase, another critical signaling molecule in conventional preconditioning pathways.^33^ For these reasons, we anticipated that 7 daily episodes of IPC would put a COX‐2‐dependent signaling cascade into effect. However, our results suggest that the chronic preconditioning stimuli in this study may have provided endothelial protection through a pathway that differs from the conventional signaling pathways of acute preconditioning. This result is actually consistent with the results reported from Depre et al^30^ where the protection from repeated episodes of IPC were not dependent on nitric oxide synthase.

The fact that the present data were acquired in healthy volunteers and in forearm conduit vessels is acknowledged as a limitation. However, the model of forearm IR injury used in the present study has been previously shown to provide reliable and relevant information regarding the effect of various agents on the development of IR‐induced endothelial dysfunction.^4,12–13,35^ The small sample size in the present study and our observed effect size for IR in the control groups, which was smaller than what we have reported previously, must also be acknowledged. However, we believe that the possibility of type I or II statistical errors is unlikely since the effect of IPC on FMD was more pronounced than what we had originally hypothesized, such that our statistical power remained high. However, the high variability in our blood flow data does not permit us to exclude the possibility of a type II error due to low statistical power.

The present study demonstrates, for the first time in humans, that repeated episodes of IPC confer significant and equipotent protection against IR‐induced endothelial dysfunction in the forearm vasculature as that observed with a single episode of IPC. This chronic preconditioning regimen does not appear to be dependent on COX‐2, which suggests that a novel signaling pathway, distinct from those known to be involved in acute preconditioning signaling, may be mediating the protective effects observed. With the inclusion of the current data, the evidence from human chronic preconditioning studies suggests that ischemia may be more effective than pharmacologic agents as a preconditioning trigger when applied in a prolonged fashion. The mechanistic differences between chronic IPC and chronic administration of agents such as nitroglycerin and HMG‐CoA reductase inhibitors have yet to be determined and are an area of future research. Elucidating the mechanisms that govern chronic forms of preconditioning will permit a better understanding of the clinical potential for this new branch of preconditioning strategies.

## References

[1] Ovize M Baxter GF Di Lisa F Ferdinandy P Garcia‐Dorado D Hausenloy DJ Heusch G Vinten‐Johansen J Yellon DM Schulz R Working Group of Cellular Biology of Heart of European Society of Cardiology Postconditioning and protection from reperfusion injury: where do we stand? Position paper from the Working Group of Cellular Biology of the Heart of the European Society of Cardiology. Cardiovasc Res. 2010; 87:406-423 20448097 10.1093/cvr/cvq129

[2] Vinten‐Johansen J Zhao ZQ Nakamura M Jordan JE Ronson RS Thourani VH Guyton RA Nitric oxide and the vascular endothelium in myocardial ischemia‐reperfusion injury. Ann N Y Acad Sci. 1999; 874:354-370 10415547 10.1111/j.1749-6632.1999.tb09251.x

[3] Laude K Beauchamp P Thuillez C Richard V Endothelial protective effects of preconditioning. Cardiovasc Res. 2002; 55:466-473 12160943 10.1016/s0008-6363(02)00277-8

[4] Kharbanda RK Peters M Walton B Kattenhorn M Mullen M Klein N Vallance P Deanfield J MacAllister R Ischemic preconditioning prevents endothelial injury and systemic neutrophil activation during ischemia–reperfusion in humans in vivo. Circulation. 2001; 103:1624-1630 11273988 10.1161/01.cir.103.12.1624

[5] Ehring T Krajcar M Baumgart D Kompa S Hummelgen M Heusch G Cholinergic and alpha‐adrenergic coronary vasomotion [corrected] with increasing ischemia–reperfusion injury. Am J Physiol. 1995; 268:H886-H894 7864216 10.1152/ajpheart.1995.268.2.H886

[6] Scarabelli T Stephanou A Rayment N Pasini E Comini L Curello S Ferrari R Knight R Latchman D Apoptosis of endothelial cells precedes myocyte cell apoptosis in ischemia/reperfusion injury. Circulation. 2001; 104:253-256 11457740 10.1161/01.cir.104.3.253

[7] Murry CE Jennings RB Reimer KA Preconditioning with ischemia: a delay of lethal cell injury in ischemic myocardium. Circulation. 1986; 74:1124-1136 3769170 10.1161/01.cir.74.5.1124

[8] Liem DA Honda HM Zhang J Woo D Ping P Past and present course of cardioprotection against ischemia–reperfusion injury. J Appl Physiol. 2007; 103:2129-2136 17673563 10.1152/japplphysiol.00383.2007

[9] Leesar MA Stoddard MF Dawn B Jasti VG Masden R Bolli R Delayed preconditioning‐mimetic action of nitroglycerin in patients undergoing coronary angioplasty. Circulation. 2001; 103:2935-2941 11413083 10.1161/01.cir.103.24.2935

[10] Walsh SR Tang TY Kullar P Jenkins DP Dutka DP Gaunt ME Ischaemic preconditioning during cardiac surgery: systematic review and meta‐analysis of perioperative outcomes in randomised clinical trials. Eur J Cardiothorac Surg. 2008; 34:985-994 18783958 10.1016/j.ejcts.2008.07.062

[11] Botker HE Kharbanda R Schmidt MR Bottcher M Kaltoft AK Terkelsen CJ Munk K Andersen NH Hansen TM Trautner S Lassen JF Christiansen EH Krusell LR Kristensen SD Thuesen L Nielsen SS Rehling M Sorensen HT Redington AN Nielsen TT Remote ischaemic conditioning before hospital admission, as a complement to angioplasty, and effect on myocardial salvage in patients with acute myocardial infarction: a randomised trial. Lancet. 2010; 375:727-734 20189026 10.1016/S0140-6736(09)62001-8

[12] Gori T Sicuro S Dragoni S Donati G Forconi S Parker JD Sildenafil prevents endothelial dysfunction induced by ischemia and reperfusion via opening of adenosine triphosphate‐sensitive potassium channels: a human in vivo study. Circulation. 2005; 111:742-746 15699265 10.1161/01.CIR.0000155252.23933.2D

[13] Gori T Di Stolfo G Sicuro S Dragoni S Lisi M Forconi S Parker JD Nitroglycerin protects the endothelium from ischaemia and reperfusion: human mechanistic insight. Br J Clin Pharmacol. 2007; 64:145-150 17324239 10.1111/j.1365-2125.2007.02864.x PMC2000627

[14] Dragoni S Gori T Di SG Sicuro S Forconi S Parker JD Folic acid does not limit endothelial dysfunction induced by ischemia and reperfusion: a human study. J Cardiovasc Pharmacol. 2005; 46:494-497 16160603 10.1097/01.fjc.0000177983.68563.d1

[15] Gori T Dragoni S Lisi M Di Stolfo G Sonnati S Fineschi M Parker JD Conduit artery constriction mediated by low flow a novel noninvasive method for the assessment of vascular function. J Am Coll Cardiol. 2008; 51:1953-1958 18482663 10.1016/j.jacc.2008.01.049

[16] Yellon DM Downey JM Preconditioning the myocardium: from cellular physiology to clinical cardiology. Physiol Rev. 2003; 83:1113-1151 14506302 10.1152/physrev.00009.2003

[17] Yellon DM Hausenloy DJ Myocardial reperfusion injury. N Engl J Med. 2007; 357:1121-1135 17855673 10.1056/NEJMra071667

[18] Lambiase PD Edwards RJ Cusack MR Bucknall CA Redwood SR Marber MS Exercise‐induced ischemia initiates the second window of protection in humans independent of collateral recruitment. J Am Coll Cardiol. 2003; 41:1174-1182 12679219 10.1016/s0735-1097(03)00055-x

[19] Dana A Baxter GF Walker JM Yellon DM Prolonging the delayed phase of myocardial protection: repetitive adenosine a1 receptor activation maintains rabbit myocardium in a preconditioned state. J Am Coll Cardiol. 1998; 31:1142-1149 9562020 10.1016/s0735-1097(98)00054-0

[20] Kuzume K Wolff RA Amakawa K Van Winkle DM Sustained exogenous administration of Met5‐enkephalin protects against infarction in vivo. Am J Physiol Heart Circ Physiol. 2003; 285:H2463-H2470 12869377 10.1152/ajpheart.00341.2003

[21] Peart JN Gross GJ Morphine‐tolerant mice exhibit a profound and persistent cardioprotective phenotype. Circulation. 2004; 109:1219-1222 14993125 10.1161/01.CIR.0000121422.85989.BD

[22] Mensah K Mocanu MM Yellon DM Failure to protect the myocardium against ischemia/reperfusion injury after chronic atorvastatin treatment is recaptured by acute atorvastatin treatment: a potential role for phosphatase and tensin homolog deleted on chromosome ten? J Am Coll Cardiol. 2005; 45:1287-1291 15837263 10.1016/j.jacc.2005.01.021

[23] Gori T Dragoni S Di Stolfo G Sicuro S Liuni A Luca MC Thomas G Oelze M Daiber A Parker JD Tolerance to nitroglycerin‐induced preconditioning of the endothelium: a human in vivo study. Am J Physiol Heart Circ Physiol. 2010; 298:H340-H345 19933412 10.1152/ajpheart.01324.2008

[24] Briguori C Colombo A Airoldi F Violante A Focaccio A Balestrieri P Paolo EP Golia B Lepore S Riviezzo G Scarpato P Librera M Bonizzoni E Ricciardelli B Statin administration before percutaneous coronary intervention: impact on periprocedural myocardial infarction. Eur Heart J. 2004; 25:1822-1828 15474697 10.1016/j.ehj.2004.07.017

[25] Pasceri V Patti G Nusca A Pristipino C Richichi G Di SG Randomized trial of atorvastatin for reduction of myocardial damage during coronary intervention: results from the ARMYDA (Atorvastatin for Reduction of MYocardial Damage during Angioplasty) study. Circulation. 2004; 110:674-678 15277322 10.1161/01.CIR.0000137828.06205.87

[26] Patti G Pasceri V Colonna G Miglionico M Fischetti D Sardella G Montinaro A Di Sciascio G Atorvastatin pretreatment improves outcomes in patients with acute coronary syndromes undergoing early percutaneous coronary intervention: results of the ARMYDA‐ACS randomized trial. J Am Coll Cardiol. 2007; 49:1272-1278 17394957 10.1016/j.jacc.2007.02.025

[27] Mannacio VA Iorio D De AV Di LF Musumeci F Effect of rosuvastatin pretreatment on myocardial damage after coronary surgery: a randomized trial. J Thorac Cardiovasc Surg. 2008; 136:1541-1548 19114204 10.1016/j.jtcvs.2008.06.038

[28] Liuni A Luca MC Gori T Parker JD Loss of the preconditioning effect of rosuvastatin during sustained therapy: a human in vivo study. Am J Physiol Heart Circ Physiol. 2012; 302:H153-H158 22003053 10.1152/ajpheart.00083.2011

[29] Shen Y‐T Depre C Yan L Park JY Tian B Jain K Chen L Zhang Y Kudej RK Zhao X Sadoshima J Vatner DE Vatner SF Repetitive ischemia by coronary stenosis induces a novel window of ischemic preconditioning. Circulation. 2008; 118:1961-1969 18936329 10.1161/CIRCULATIONAHA.108.788240 PMC2634862

[30] Depre C Park JY Shen YT Zhao X Qiu H Yan L Tian B Vatner SF Vatner DE Molecular mechanisms mediating preconditioning following chronic ischemia differ from those in classical second window. Am J Physiol Heart Circ Physiol. 2010; 299:H752-H762 20581088 10.1152/ajpheart.00147.2010 PMC2944496

[31] Cohen MV Yang XM Downey JM Conscious rabbits become tolerant to multiple episodes of ischemic preconditioning. Circ Res. 1994; 74:998-1004 8156647 10.1161/01.res.74.5.998

[32] Papadopoulos CE Karvounis HI Parharidis GE Louridas GE Multiple episodes of ischemic preconditioning are not associated with loss of benefit: preliminary clinical experience. Can J Cardiol. 2005; 21:1291-1295 16341299

[33] Bolli R Preconditioning: a paradigm shift in the biology of myocardial ischemia. Am J Physiol Heart Circ Physiol. 2007; 292:H19-H27 16963615 10.1152/ajpheart.00712.2006 PMC3242363

[34] Bolli R Shinmura K Tang X‐L Kodani E Xuan Y‐T Guo Y Dawn B Discovery of a new function of cyclooxygenase (Cox)‐2: Cox‐2 is a cardioprotective protein that alleviates ischemia/reperfusion injury and mediates the late phase of preconditioning. Cardiovasc Res. 2002; 55:506-519 12160947 10.1016/s0008-6363(02)00414-5 PMC3242376

[35] Liuni A Luca MC Gori T Parker JD Rosuvastatin prevents conduit artery endothelial dysfunction induced by ischemia and reperfusion by a cyclooxygenase‐2‐dependent mechanism. J Am Coll Cardiol. 2010; 55:1002-1006 20202516 10.1016/j.jacc.2009.11.046

